# Automated Quantification of sTIL Density with H&E-Based Digital Image Analysis Has Prognostic Potential in Triple-Negative Breast Cancers

**DOI:** 10.3390/cancers13123050

**Published:** 2021-06-18

**Authors:** Jeppe Thagaard, Elisabeth Specht Stovgaard, Line Grove Vognsen, Søren Hauberg, Anders Dahl, Thomas Ebstrup, Johan Doré, Rikke Egede Vincentz, Rikke Karlin Jepsen, Anne Roslind, Iben Kümler, Dorte Nielsen, Eva Balslev

**Affiliations:** 1 Department of Applied Mathematics and Computer Science, Technical University of Denmark, 2800 Kgs. Lyngby, Denmark; lgv@visiopharm.com (L.G.V.); sohau@dtu.dk (S.H.); abda@dtu.dk (A.D.); 2 Visiopharm A/S, 2970 Hørsholm, Denmark; teb@visiopharm.com (T.E.); jdh@visiopharm.com (J.D.); 3 Department of Pathology, Herlev and Gentofte Hospital, 2730 Herlev, Denmark; elisabeth.ida.specht.stovgaard@regionh.dk (E.S.S.); rikke.egede.vincentz.02@regionh.dk (R.E.V.); rikke.karlin.jepsen@regionh.dk (R.K.J.); anne.roslind@regionh.dk (A.R.); Eva.Balslev@regionh.dk (E.B.); 4 Department of Oncology, Herlev and Gentofte Hospital, 2730 Herlev, Denmark; Iben.Kumler@regionh.dk (I.K.); dorte.nielsen.01@regionh.dk (D.N.)

**Keywords:** deep learning, digital pathology, image analysis, prognostic biomarker, survival analysis, triple-negative breast cancer, tumor microenvironment (TME), tumor-infiltrating lymphocytes

## Abstract

**Simple Summary:**

Around 15% of breast cancer patients are diagnosed as triple-negative (TNBC), which have significantly lower 5-year survival rates (77%) than other types of breast cancer (93%). Our study aimed at developing an image analysis-based biomarker to assess how the immune system interacts with the tumor and investigate the potential added value of stromal tumor-infiltrating lymphocytes (sTIL) for the prognosis of overall survival compared to the manual approach. In a large retrospective cohort of 257 patients, we found that our fully automated hematoxylin and eosin (H&E) image analysis pipeline can quantify sTIL density showing both high concordance with manual scoring and association with the prognosis of patients with TNBC. It also overcomes natural limitations of manual assessment that hinder clinical adoption of the immune biomarker. We conclude that sTIL scoring by automated image analysis has prognostic potential comparable to manual scoring and should be further investigated for future use in a clinical setting.

**Abstract:**

Triple-negative breast cancer (TNBC) is an aggressive and difficult-to-treat cancer type that represents approximately 15% of all breast cancers. Recently, stromal tumor-infiltrating lymphocytes (sTIL) resurfaced as a strong prognostic biomarker for overall survival (OS) for TNBC patients. Manual assessment has innate limitations that hinder clinical adoption, and the International Immuno-Oncology Biomarker Working Group (TIL-WG) has therefore envisioned that computational assessment of sTIL could overcome these limitations and recommended that any algorithm should follow the manual guidelines where appropriate. However, no existing studies capture all the concepts of the guideline or have shown the same prognostic evidence as manual assessment. In this study, we present a fully automated digital image analysis pipeline and demonstrate that our hematoxylin and eosin (H&E)-based pipeline can provide a quantitative and interpretable score that correlates with the manual pathologist-derived sTIL status, and importantly, can stratify a retrospective cohort into two significant distinct prognostic groups. We found our score to be prognostic for OS (HR: 0.81 CI: 0.72–0.92 *p* = 0.001) independent of age, tumor size, nodal status, and tumor type in statistical modeling. While prior studies have followed fragments of the TIL-WG guideline, our approach is the first to follow all complex aspects, where appropriate, supporting the TIL-WG vision of computational assessment of sTIL in the future clinical setting.

## 1. Introduction

The host immune system and interactions in the tumor microenvironment (TME) play an important role in clinical outcomes for patients with triple-negative breast cancer (TNBC) [1,2,3]. TNBC is an aggressive and difficult-to-treat cancer type that represents approximately 15% of all breast cancers [4]. It is defined by a lack of estrogen and progesterone hormone receptors (ER/PR) and expression of human epidermal growth factor receptor 2 (HER2), i.e., common treatment options are not very effective, resulting in a lower 5-year survival rate (77%) than other types of breast cancer (93%) [5,6].

Recently, stromal tumor-infiltrating lymphocytes (sTIL) have resurfaced as a strong prognostic biomarker for overall survival (OS) [7,8,9,10], and guidelines for manual assessment have been proposed [11] to standardize reporting, increase reproducibility, and improve clinical adoption [12,13]. Nevertheless, the manual assessment has innate limitations [14] that hinder clinical adoption. These include human limitations such as inter-reader variability, bias, and limits of the routine diagnostic laboratory such as time and staff constraints, especially in remote and under-resourced settings. The International Immuno-Oncology Biomarker Working Group (TIL-WG) has therefore envisioned that computational assessment of sTIL could overcome the limitations of manual assessment and recommended that any algorithm should follow the manual guidelines where appropriate [15]. However, to the best of our knowledge, no published computational approach exists that follows all the key steps of the TIL-scoring guideline.

sTIL consists of a pool of immune cell types found in the TME such as cytotoxic CD8+ T-cells, natural killer (NK) cells, macrophages, T-helper cells, and immune-suppressing B-cells and regulatory CD4+ T-cells [16,17]. T-cells make up the majority of TILs in breast cancer [18]. It has a long history as a prognostic biomarker (more than 100 years) [19], but its clinical validity for early-stage TNBC was only recently well-established through level 1b evidence [20,21,22]. Incorporating sTILs into standard clinical practice is now endorsed by multiple international clinical standards since 2019 (St. Gallen Breast Cancer Expert Committee [12], World Health Organization (WHO) [23], and ESMO [24]). The guidelines to manually score sTIL status is proposed by the TIL-WG, and briefly, scored as the area of tumor-associated stroma occupied by TILs estimated as a percentage of total tumor-associated stromal area, where areas of necrosis, ductal and lobular carcinoma in situ (DCIS/LCIS), and normal breast tissue are excluded [25].

Most studies of computational TILs have employed patch- or object detection-based approaches [26,27,28,29] with manual region outlining as part of the pipeline [30]. Some of these also used multiplexed immunofluorescence (mIF) [31] or immunohistochemistry (IHC) [32,33] to classify cells as lymphocytes. All existing studies proposing H&E-based algorithms rely on only manual H&E ground truth annotations to train their model even though the manual human limitations have shown inconsistencies in this task [14]. None of these studies capture all the concepts of stromal and intratumoral TILs and account for confounding morphologies specific to different tumor sites, subtypes, and histologic patterns as envisioned by the TIL-WG [15]. Another unanswered question is the objective of an automated approach, i.e., whether the performance should be measured as the concordance between manual and automated sTIL status, the clinical outcome of the patient, or a mix of both [34].

In this study, we present a fully automated digital image analysis pipeline that integrates key aspects of the manual guideline to compute a prognostic biomarker for TNBC patients. Our approach combines both cell- and tissue-level information from whole slide images (WSIs) in both creation of ground truth annotations and during inference, which enables a robust approach that can be employed on routine H&E-stained slides. We show the existence of human inter-observer variability in the ground truth generation, and we propose to use combinatory IHC to generate more objective ground truth for both cell- and tissue-level models. We demonstrate that our H&E-based pipeline can provide a quantitative and interpretable score that correlates with the manual pathologist-derived sTIL status, and importantly, has the potential to show the prognostic implications of the sTIL status in a retrospective cohort of TNBC patients in a manner comparable to manual scoring.

## 2. Materials and Methods

### 2.1. Data Sources and Study Population

We used a cohort of patients operated for primary TNBC at Herlev and Hillerød Hospitals, Denmark, between 1 January 2004 and 31 December 2010, and who had freshly cut and stained H&E full tumor slides available. The exclusion criteria were neoadjuvant chemotherapy, previous malignancy within the past 5 years prior to diagnosis, recurrence of previous breast cancer, bilateral/multifocal breast cancer, and tumors with only microinvasion. If previous HER2 analysis had not been performed, this was conducted at the time of inclusion in the study, and patients with HER2 overexpression were excluded. A total of 262 eligible patients had freshly cut and stained H&E-stained slides from original tumor blocks from primary surgery available for analysis (a flowchart of in- and exclusion in the study can be seen in Supplementary Figure S1). Clinical information was gathered from the patient journals and/or pathology reports. A follow-up was completed on 1 July 2019. All clinical data were stored and processed at the Pathology Department, Herlev, and Gentofte Hospital, and no third party had access to data with patient information. See Supplementary Table S1 for an overview of included patients.

Patients in the inclusion period received standard chemotherapy regimens and radiation therapy if indicated. Chemotherapy regimes varied somewhat over time, as standard chemotherapy treatment in Denmark consisted of cyclophosphamide, epirubicin, and 5-fluorouracil (5-FU) from 2004 to 2007, and epirubicin, cyclophosphamide, and docetaxel from 2007 to 2010.

The H&E-staining was performed according to a well-established protocol also used in daily diagnostics at the Department of Pathology, Herlev and Gentofte Hospital, Denmark. The 4 µm slides were sectioned from formalin-fixed, paraffin-embedded (FFPE) tumor blocks and mounted on glass slides. The tissue was then deparaffinized in Tissue Clear (SAKURA Tissue Tek) and alcohol, washed with water and stained with Mayers hematoxylin (pH 2.7) and eosin (diluted with 70% alcohol), and finally treated with 99% alcohol before cover-slipping. Staining procedures varied minimally over the inclusion period, and for the digital pipeline, only freshly sectioned and stained slides were used following the procedure outlined above.

For the model development, we used only fully anonymized H&E-stained slides of TNBC tumors from Herlev Hospital, as well as publicly available slides from the TCGA-BRCA database.

The evaluation of tumor-infiltrating lymphocytes in TNBC was approved by the Danish Ethics Committee (project number H-15015306). The material used in the study was previously obtained for clinical purposes. At the time of collection, patients were informed that the material could be used for research purposes unless they registered actively in The Danish Registry for Use of Tissue. No patients included in this study had registered there.

### 2.2. Fully Automated Image Analysis Pipeline Design

**Figure 1:**
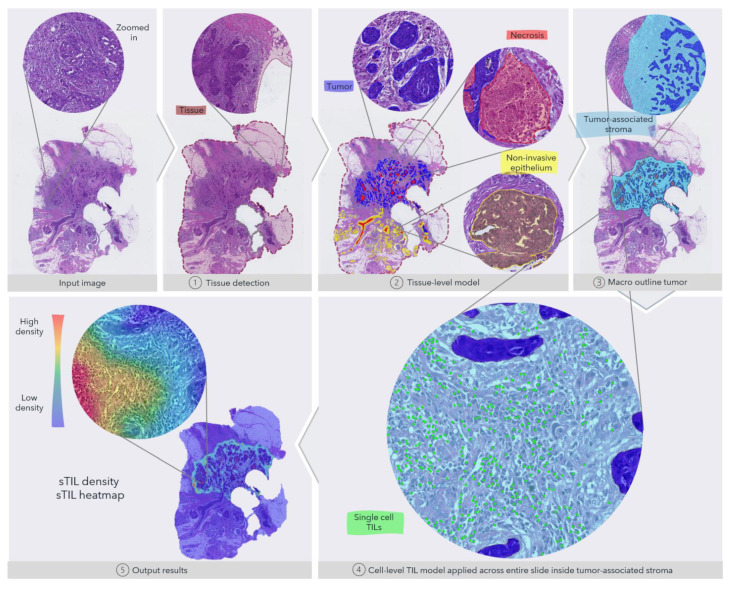
Overview of the fully automated image analysis pipeline. The input data are the scanned WSI of a TNBC patient, which is then analyzed by multiple steps. First, the tissue (dark red) is recognized from the glass to limit the analysis to only the relevant part of the scanned slide. Secondly, the tissue-level model classifies slide regions into tumor tissue (blue), non-invasive epithelium (yellow), and necrotic regions (red). In the third step, the macro-outline of the tumor is approximated, and then tumor-associated stroma and margin (turquoise) are defined. Cells across the entire sample in the tumor-associated stroma are classified as TILs (green) or not, and finally, the sTIL density and heatmap can be outputted for review.

In order to support a fully automated image analysis, we developed multiple steps into a combined algorithm: (1) we trained a convolutional neural network (CNN) to detect the tissue from the background glass slide at 5X magnification to limit the analysis to only the relevant regions; (2) a second tissue-level CNN at 10X to segment tumor, necrosis and non-invasive epithelial (normal, pre-invasive lesions); (3) an object-based density analysis of tumor regions to estimate the macro outlining of the entire tumor, hence defining the tumor-associated stroma; (4) a third cell-level CNN at 20X to detect and classify cells as TILs (mononuclear immune cells); and finally, (5) output result and local density calculation (heatmap) to quantify and visualize extracted information from the tissue- and cell-level models. The full pipeline is shown in Figure 1. All digital image analysis steps were developed and performed with the Visiopharm platform (Visiopharm A/S, Hørsholm, Denmark).

We trained all CNNs with a VGG-based encoder pre-trained on ImageNet [35], where the tissue- and cell-level models use DeepLabV3 [36] and U-Net [37] inspired decoders, respectively. We applied random color augmentation (brightness, contrast, hue, and saturation), H&E stain augmentation [38], and spatial transformation (rotation, flipping). See Section 2.3 for more information on the dataset development used for these models.

To define the tumor-associated stroma, we evaluated the local accumulated tumor area using a fixed circular kernel (radius = 750 µm) combined with morphological operations (closing/opening). The approach was designed to mimic how the pathologist would draw the macro outline of the entire tumor. We included a margin of 250 µm from the border of the tumor into the surrounding stroma. This approximation of the margin aligns with the TIL-WG guideline on including the invasive margin.

To obtain the cellular density of sTIL, we applied the cell-level TIL model across the entire macro-tumor and excluded detected TILs within regions of necrosis, a central hyalinized scar in the tumor core, tumor, and within 150 µm proximity of non-invasive epithelial to avoid dense lymphatic aggregates surrounding these regions.

Lastly, we calculated the sTIL density as the number of TILs within the tumor-associated stroma per mm^2^. We also calculated the local density with a fixed circular kernel (radius = 200 µm) and visualized this as a heatmap to provide both a quantitative and visual estimate of the sTIL heterogeneity for a reviewing pathologist.

### 2.3. Cell and Tissue-Level Model Development

**Figure 2:**
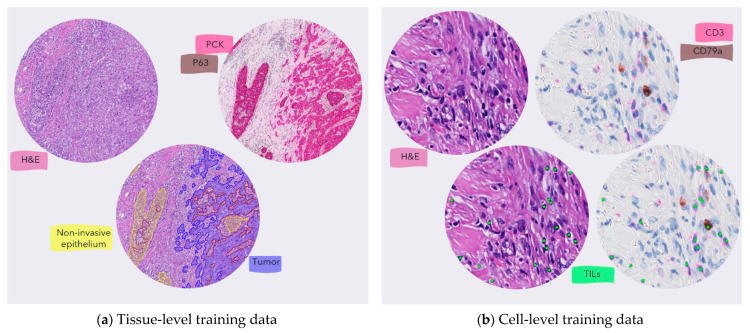
The process to generate objective training data. (**a**) The training annotations for the tissue-level model were generated using IHC when available. For the images from the TCGA-BRCA, the annotations were manually generated by a pathologist. (**b**) the TILs training annotations were generated as center-dot labels on cells that were either CD3 or CD79a positive to make sure that all mononuclear immune cells were included as stated by the TIL-WG guideline.

To obtain robust performance of both our tissue- and cell-level models, we developed them using an IHC-guide annotation scheme on a holdout set (*n* = 21 patients) from the Herlev cohort (see Figure 2) supplemented by expert pathologist annotations for the tissue-level model on a subset (*n* = 55 images) of the TCGA-BRCA dataset.

For the tissue-level model, we created new consecutive serial sections stained with H&E and pan-cytokeratin (PCK; clone AE1/AE3, DAKO Omnis) + P63 (clone DAK-P63, DAKO Omnis), respectively in the holdout set from Herlev. To generate the training data, we digitally aligned two slides using an affine registration algorithm (Tissuealign, Visiopharm A/S, Hørsholm, Denmark) and iteratively selected FOVs manually to maximize the variation in morphology of stroma, tumor, necrotic, and non-invasive regions. To increase the robustness of the model and the variation in the training data, we also included manually annotated slides from TCGA-BRCA and used the same iterative process until we saw no further performance increase on a small holdout set of the development data. We conducted the final training and validation of the tissue-level model on a ground truth dataset (*n* = 76 images) verified by a single pathologist (ES) before including it in the full pipeline for testing.

For the cell-level model, we only used a holdout set from Herlev as we created new sections that were first stained with H&E, then scanned, followed by removal of H&E with re-staining of a chromogenic IHC protocol (CD3 (clone F7.2.38, DAKO) and CD79a (clone JCB117, DAKO Omnis)) to highlight all mononuclear immune cells (lymphocytes and plasma cells). After digitalization, we aligned the images of the same sections as above and used a similar iterative approach to select FOVs to maximize the variation of low-, mid-, and high-density lymphocyte regions in both close and distance proximity to tumor regions. To the best of our knowledge, we are the first to apply this approach to obtain ground truth annotations for the detection and classification of TILs in H&E-stained sections. We trained and validated the final cell-level model on a ground truth dataset (*n* = 12 images) spanning 69 FOVs and 7277 individual lymphocytes and plasma cells. This dataset was also verified by a single pathologist (ES) reviewing all annotations with both H&E and IHC staining side-by-side.

As we deemed the cell-level model most critical to the full analysis pipeline, we conducted further testing against three expert pathologists before including it in the full pipeline, see Section 2.4 below.

### 2.4. Inter-Reader Variability and Validation of the Cell-Level Model

We obtained the validation set and investigated the following three key aspects; (1) the effect of having IHC available on manual recognition of a cell as a lymphocyte or not, (2) the inter-reader variability between manual readers using H&E only, and (3) the analytical performance of the cell-level TIL model. This was performed by having three pathologists mark and count sTILs. One pathologist (ES) with H&E aligned with IHC and two (RV and RJ) with H&E only to mimic the clinical setting. We used full slide images (*n* = 4) that were not part of the development data, where we preselected a total of 12 FOVs spanning a range of low, mid, and high-density TIL regions in intertumoral stroma varying range of proximity to tumor regions. The pathologist with access to H&E and IHC used the Visiopharm platform (Visiopharm A/S, Hørsholm, Denmark) to align the two images, so information from both could be displayed at the same time at a cellular level. The pathologists with access to only H&E used the Concentriq platform (Proscia Inc., Philadelphia, MA, USA) to mark cells as sTILs, which then could be imported to the Visiopharm platform for further analysis.

### 2.5. Manual Biomarker Assessment

To obtain the manual sTIL status, we used H&E slides from two FPPE tumor blocks, if available, and averaged the score or a single slide if only one block was available. Either the original H&E slides from diagnostics following primary surgery were used, or two new 4 micrometer slices were cut and stained with H&E following routine procedures. The sTIL evaluation followed guidelines published by the TIL-WG [25]. Three pathologists (ES, AR, and EB) evaluated 204 cases, and the remaining cases were evaluated by a single pathologist (ES) with a consensus reached with the other two pathologists in difficult cases. We used the manual sTIL status as a continuous variable when possible and with a cutpoint of >10% [21,39,40,41].

### 2.6. Statistical Analysis

We used overall survival (OS) as the primary endpoint for prognostic analysis, defined as the time from primary surgery until death from any cause with censoring at the last visit date. We also included relapse-free survival (RFS), defined as the time from primary surgery to local or distant relapse with censoring at death or date of the last visit, as the secondary endpoint.

We applied the Kaplan–Meier method [42] to estimate OS and RFS, and Cox proportional hazard models [43] to quantify the hazard ratio (HR) for the effects of biomarker groups (continuous or with distinct cut-offs). For continuous variables, we divided the manual sTIL with 10, and the sTIL Density with 300, so the HRs given represent differences of increments of 10 and 300, respectively.

The multivariate analysis included age (≥50 vs. <50 years), tumor size (≤2 vs. >2 cm), number of lymph node metastases at primary surgery (0 vs. 1–3, 0 vs. ≥4), tumor type (ductal vs. lobular, ductal vs. other). Only cases with complete data were included in the multivariate analysis.

We conducted all statistical analyses in the R (version 4.0.3).

## 3. Results

### 3.1. Automatic sTIL Density Is Associated with Improved Overall Survival

**Figure 3:**
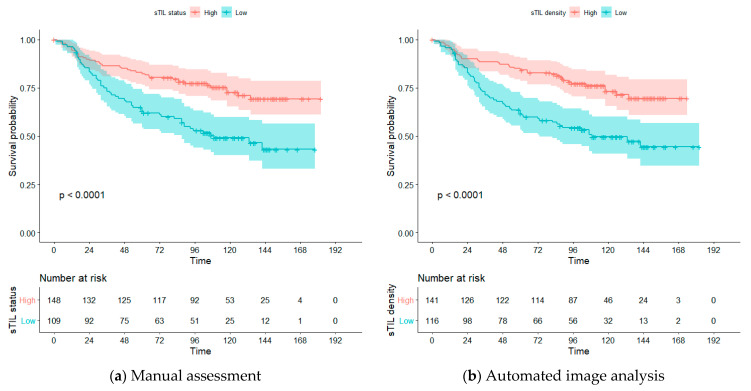
Overall survival estimated by Kaplan–Meier analysis. (**a**) Stratification of patients into high (red) and low (blue) group using a cutpoint of >10% on the manual sTIL status. (**b**) stratification of patients into a high (red) and low (blue) group using a cutpoint of 470 sTIL/mm^2^ for the automated sTIL density.

Manually assessed sTIL is known to be associated with prognosis in TNBC patients [21,44], often stratified into two prognostic groups: high and low sTIL status [21,39,40]. To be able to investigate if the sTIL density score is similarly associated with OS, we also stratified the patient cohort into two groups: high and low sTIL density by using maximally selected rank statistics [45] for cutpoint selection of our automated approach. We found an optimal cutpoint of 470 sTIL/mm^2^ and used this to estimate OS according to the Kaplan–Meier method, and compared the results to the manual sTIL status with cutpoint > 10% [21,39,40], see Figure 3. For the included cohort, both manual sTIL status and sTIL density stratified the patients significantly into two distinct prognostic groups (*p* < 0.0001).

#### 3.1.1. Univariate Analysis

To further compare our method’s association with OS, we conducted a univariate analysis on both manual sTIL status and sTIL density as a continuous variable (see Table 1). Higher sTILs scores evaluated both automatically and manually were associated with significantly prolonged OS. Every 10% or 300 sTILs/mm^2^ increase in the biomarker score results in ~20% decrease in risk of death for manual (HR: 0.81 CI: 0.71–0.93) and automated score (HR 0.82 CI: 0.72–0.93), respectively. Neither of the methods was significant for RFS, with only the nodal status being significantly associated with RFS (see Table 1). Most noticeably, the univariate analysis confirmed the same significant and independent prognostic value of automated sTIL density and manual sTIL assessment as a continuous variable.

#### 3.1.2. Multivariate Analysis

To investigate the added prognostic information of sTIL density versus sTIL status to standard clinical prognostic factors, we used multivariate analysis on both OS and RFS variables (see Table 2). sTIL density was still found to be prognostic for OS (HR: 0.81 CI: 0.72–0.92 *p* = 0.001) independent of age, tumor size, nodal status, and tumor type. The same was observed for manual sTIL status (HR: 0.79 CI: 0.68–0.91 *p* = 0.001). For RFS, both methods were found to be significant.

### 3.2. Cell-Level TIL Model Correlates with Manual Expert Pathologists

**Figure 4:**
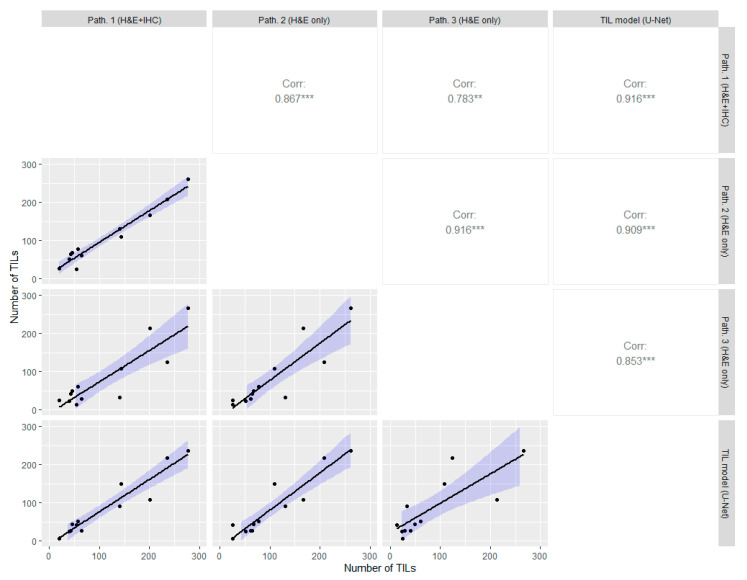
Inter-method variability of cell-level discrimination of TILs between the pathologist with both H&E and IHC, the two pathologists with only H&E, and our image analysis approach on a holdout test set. The lower left of the diagonal shows the correlations plot, and the upper right shows the Spearman correlation coefficient for each comparison. The asterisks ** (*p* ≤ 0.01) and *** (*p* ≤ 0.001) indicate the significance levels of the statistical correlation test.

Previous studies have shown inter-reader variability for identifying individual sTILs in H&E [14,46]. Therefore, a key part of the fully automated pipeline is to be able to count the correct number of sTILs. To determine the degree of inter-reader variability and the analytical validation of the cell-level TIL model, we used the data described in Section 2.3, where we also applied the TIL model to the same regions to measure the agreement. The results are shown in Figure 4 of the correlation between the approaches. The TIL model had a high correlation with all three pathologists, especially the pathologist with access to both H&E and IHC CD3 + CD79a (Spearman correlation coefficient r_s_ = 0.916). Moreover, the inter-reader agreement between the pathologist was also high, but with the lowest correlation between the pathologist with access IHC and pathologist 3 (r_s_ = 0.783). The lowest correlation to the TIL model was seen between pathologist 3 (r_s_ = 0.853), where the pathologist counted fewer TILs in many cases. Overall, we observed an inter-reader variability between the expert pathologists and that the TIL model had the highest correlation with the pathologist who had access to the same information (H&E + IHC) as the TIL model was trained against.

### 3.3. Automatic sTIL Density Correlates with Manual sTIL Assessment on Full Section H&E Slides

**Figure 5:**
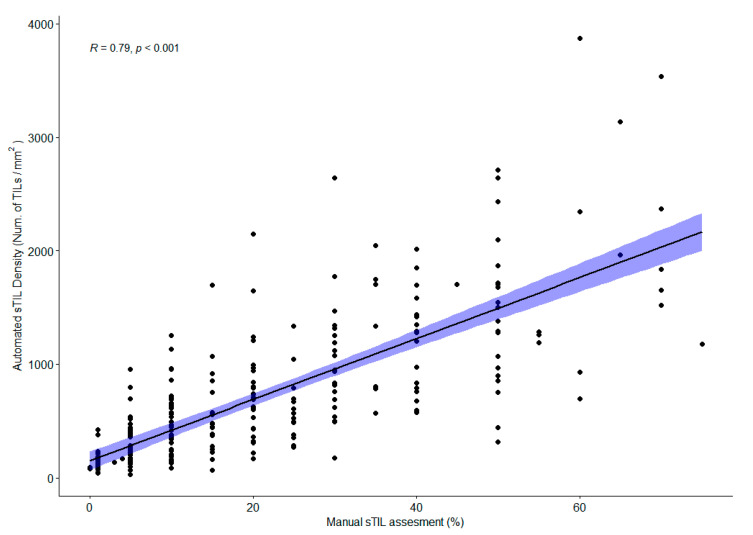
Correlation between manual sTIL assessment and automated sTIL density.

When scaling sTIL scoring up to the full tissue section, the manual assessment score is prone to many pitfalls [14] even though guidelines are followed. To validate the full automated analysis pipeline, we used Spearman correlation to test if there is a significant linear relationship between the manual sTIL assessment score (see Section 2.5) and the automatic sTIL density output from our approach, see Figure 5. We observed a significantly high correlation (r_s_ = 0.79, *p* < 0.001) between the two methods. As expected, we did not see a perfect correlation as our method uses the computed sTILs per mm^2^, whereas the manual scoring guideline is an estimate of area coverage by sTIL. We also observed larger disagreement for higher sTIL scores comparable to the inter-pathologist agreement for manual scoring whole section cases [47]. The result is comparable to the variance observed between pathologists scoring sTIL [14,47].

We found a total of 50 discrepant cases between low and high sTIL groups using the cutpoints for each method. At this specific cutpoint, this binary classification corresponds to a sensitivity and specificity of 81.2% and 80.5%, respectively (22 false positives and 28 false negatives). To understand these discrepant cases more, we looked at the manual score and image analysis quality. For 39 of the discrepant cases, the manual score was obtained as a consensus between 3 pathologists. The remaining 11 cases were scored by a single pathologist. Twenty-eight cases were scored >10% manually but are below the cutpoint for the automated method. For these, the average manual sTIL status is slightly above the cutpoint (µ = 21%) with an average standard deviation between pathologists of 5%, and the average sTIL density is 310 cells/mm^2^. For the other scenario, where 22 cases were scored ≤10% but were above the cutpoint for the automated method, the manual sTIL status was 10% for 82% of these cases (µ = 8.6%) with an average standard deviation between pathologist of 2%. The automated sTIL density of these cases is 725 cells/mm^2^. For 47 of the discrepant cases (94%), both scores from the manual and automated method were around their respective cutpoints, and we consider these within the expected discrepancy around cutpoints. The last three cases all had manual sTIL > 30% but were below the automated cutpoint. One case had a sectioning artifact resulting in a lower automated score. The two others had high lymphocyte infiltration along the invasive margin but almost no sTILs in the central tumor-associated stroma. The discrepancy might result from how the contribution from the two compartments was averaged as the automated method does not treat the two compartments (invasive margin and tumor-associated stroma) equally but averages the density across all tumor-associated stroma.

## 4. Discussion

In this study, we designed a digital image analysis pipeline that joins several algorithmic steps, including a tissue-level segmentation model and a cell-level TIL model that combined adhere to the manual scoring guideline by the TIL-WG. We demonstrated how our sTIL density score is independently prognostically significant for OS, similar to manual sTIL status on whole sections. Furthermore, the automatic score stratifies patients in low- and high-sTIL density groups that are highly associated with OS and correlate highly with the manual sTIL assessment. Our study shows for the first time that sTIL density in TNBC can reliably be assessed by a fully automatic deep learning pipeline.

Compared to prior attempts to apply image analysis for computational assessment of sTIL, such as patch- [26], object- [28,29], or segmentation-based methods [27,48], our study incorporates all parts of the TIL-WG guideline; from discriminating tissue from glass, and excluding necrotic regions and inflammation related to the non-invasive epithelium, such normal glands and DCIS/LCIS. A recent study [33] investigated several aspects of computational TIL assessment for prognosis in TNBC. To find the optimal compartment (margin, tumor-associated stroma, etc.), they used manual annotations and found no difference in the various regions. To investigate the immune cell population that is optimally for prognostic biomarker assessment, they used IHC for CD3, CD8, and FOXP3, and again found that all subtypes of markers correlate with survival. These observations are in line with ours as we do not discriminate between invasive margin and tumor-associated stroma but simply perform a combined assessment of the two compartments. Similarly, we do not discriminate between the immune cell subtypes but quantify all mononuclear immune cells as one class as stated by the TIL-WG guideline. These observations indicate that manual region annotations and immune cell subtypes are not necessary to obtain a prognostic immune-related biomarker for TNBC.

Recent studies have also shown the benefit of combining tissue- and cell-level deep learning models to interrogate the TME in breast cancer, such as the local TIL infiltration around DCIS structures [49], or engineering hundreds of features from these models to predict molecular signatures [50]. Our results align well with the benefits of having both multi-level analyses. In contrast to these studies, we focus on a single proven biomarker, and we sought to translate the manual guideline into a computational approach that could be performed by a computer. This can be combined with other biomarkers such as the tumor stroma ratio (TSR) [51] directly from the same H&E section, which also is associated with survival when calculated computationally on tissue microarrays (TMAs) [52], or with IHC markers such as the expression of programmed death-ligand 1 (PD-L1) [53].

To not be limited by expensive and subjective expert annotations in the development data used in this and future studies, we also rigorously focused on an objective approach to generate ground truth data that is scalable at both tissue- and cell-level. Other related applications also used similar IHC techniques to transfer annotations to H&E. Tellez et al. [38] used PHH3 to guide annotations of mitotic cells in breast cancer tissue, Bulten et al. used P63 and CK8/18 as the reference standard for a CNN to segment epithelium in prostate cancer [54], and Valkonen et al. [55] automatically transferred CD45 to an H&E slide to segment leukocytes in papillary thyroid carcinoma. Similar to ours, these methods also involve a manual step in the process. However, we use it to generate tissue- and cell-level annotations and show that this technique works for guiding annotations of all relevant mononuclear immune cells in breast cancer.

Our approach allows us to investigate and quantify the TME for a specific cellular biomarker across the entire WSI image. Hence, it overcomes the limiting constraints of manual reading as counting all cells and measuring precise stromal area in samples with complex tumor patterns is intractable to perform for a human, e.g., related to the heterogeneity in sTIL distribution [14]. Even though small differences exist in the averaging compartments between our method and the TIL-WG guideline, the sTIL density shows similar potential as a prognostic biomarker as the manual assessment for the investigated cohort. These findings also confirm previous studies in breast cancer, in which sTIL assessment is found to be associated with improved prognosis [21,44]. One of the sources for variability in manual scoring is the adherence to the guideline definition [14]. Using a computational approach that adheres to that definition increases the standardization for scoring TNBC patients, while it also shows similar concordance to the clinical outcome of those patients.

Our study also has several limitations. First, even though our models show good generalizability on the retrospective cohort (*n* = 480 WSIs), we developed them on a limited number of cases. This means that the models might not perform optimally on another study cohort from a different site with a distributional shift in, e.g., preanalytical protocols, staining protocol, or scanner type [56,57]. Future development of our approach should extend the development dataset of both tissue- and cell-level models to be multi-institutional, covering the innate variability of the above-mentioned factors.

The cutpoint for the low- and high-sTIL density also has limitations as it was found within the single study cohort. As we used the biomarker as a continuous variable in the multivariate analysis for OS, this should not affect the evidence of our methods’ association to improved prognosis. The discrepancy at the binary cutpoint between the manual and automated approach should also be compared to the variability of manual scoring (intraclass correlation coefficients of 0.77–0.94 for discrete cut-off values) [14]. However, in future validation, the optimal cutpoint should be investigated further and tested on an independent cohort. In general, new emerging biomarkers must be co-developed with a digital image analysis tool to ease the clinical adoption by pathologists. By doing so, clinicians simultaneously learn about the biomarker and familiarize themselves with the pros (and cons) of quantifying it using machine learning (ML)-based scoring approaches. Hence, the clinical validation will become a combination of the biomarker and automated scoring method providing a combined computational biomarker, and not just a digital tool add-on after years of manually scoring the biomarker. With the current pace of advancement in ML for healthcare, it will also become instrumental that existing clinicians and future generations of physicians obtain formal training in computational approaches so they can better assess the clinical needs, advice on how it is best integrated into their workflow, and perform the critical appraisal of the performance of ML-based systems [58]. All this to ensure the added value in day-to-day clinical decision making.

Even though our analytical validation of the TIL model shows a high correlation between our approach and the expert pathologist, this step of the algorithm is critical to the validity of the full pipeline. There are recent efforts by regulatory instances to develop and provide the dataset for validating exactly this kind of computational step [46]. We recommend that such efforts might be supplemented by our annotation approach to generate a more objective ground truth for estimating the density of sTIL in breast cancer, so the reliance on large-scale pathologist annotation is limited while mitigating variability in the process.

Should the automated approach then completely replace the manual sTIL assessment? No. The automated approach might be faster and more reproducible in many aspects but also has several limitations, as discussed above. We recommend using our approach as another tool in the pathologist toolbox to help increase reproducibility and handle key factors such as sTIL heterogeneity by automatically computing objective counts and area metrics recognized by the models. This is also the recommendation from the TIL-WG [15]. As the diagnostic responsibility resides with the pathologist, these metrics need to be presented quantitatively and visually for manual review and sign-off. Future development of our approach could therefore extend to investigate the impact of a combined setup of a pathologist using a computational method on the clinical outcome of the patient.

## 5. Conclusions

We demonstrated in a large retrospective cohort that a fully automated H&E image analysis pipeline could quantify sTIL density showing both high concordance with manual scoring and association with the prognosis of patients with TNBC. While prior studies have followed fragments of the TIL-WG guideline, our approach follows all complex aspects where appropriate supporting the TIL-WG vision of computational assessment of sTIL in the future clinical setting.

## Figures and Tables

**Table 1:** Univariate analysis of the included clinical parameters and biomarkers. ^1^ Manual score is in increments of 10. ^2^ sTIL density is continuous but normalized to increments of 300 sTILs/mm^2^.

Variable	HR (95% CI)			
	OS	*p*	RFS	*p*
Age	3.37 (1.75–6.49)	<0.001	1.83 (0.96–3.52)	0.068
Nodal status				
1–3	1.61 (1.01–2.55)	0.043	2.04 (1.16–3.57)	0.013
≥4	4.37 (2.57–7.43)	<0.001	4.33 (2.20–8.51)	<0.001
Tumor size	1.55 (1.00–2.41)	0.049	1.69 (0.98–2.93)	0.060
Tumor type				
Ductal vs. lobular	4.21 (1.32–13.44)	0.015	4.07 (0.98–16.94)	0.053
Ductal vs. other	0.95 (0.58–1.55)	0.826	0.74 (0.38–1.42)	0.367
sTIL status (manual) ^1^	0.81 (0.71–0.93)	0.002	0.89 (0.77–1.02)	0.090
sTIL density (auto) ^2^	0.82 (0.72–0.93)	0.002	0.87 (0.75–1.02)	0.085

**Table 2:** Multivariate analysis: ^1^ Manual score is in increments of 10. ^2^ sTIL Density is continuous but normalized to increments of 300 sTILs/mm^2^.

Method	Overall Survival			Relapse Free Survival		
	HR	95% CI	*p*-Value	HR	95% CI	*p*-Value
sTIL (manual) ^1^	0.79	0.68–0.91	0.001	0.84	0.71–0.99	0.037
Tumor Size	1.44	0.92–2.25	0.115	1.57	0.89–2.75	0.117
Age	2.96	1.52–5.77	0.001	1.72	0.88–3.35	0.112
Nodal status						
1–3	1.92	1.20–3.07	0.007	2.23	1.26–3.95	0.006
≥4	4.52	2.61–7.84	<0.001	4.42	2.19–8.90	<0.001
Tumor type						
Ductal vs. lobular	1.79	0.55–5.84	0.335	1.73	0.40–7.46	0.461
Ductal vs. other	0.91	0.55–1.51	0.718	0.74	0.38–1.45	0.384
sTIL density (auto) ^2^	0.81	0.72–0.92	0.001	0.86	0.75–1.00	0.047
Tumor Size	1.43	0.91–2.24	0.124	1.56	0.89–2.75	0.122
Age	3.02	1.55–5.90	0.001	1.76	0.90–3.43	0.099
Nodal status						
1–3	1.91	1.19–3.07	0.007	2.22	1.25–3.92	0.006
≥4	4.12	2.40–7.08	<0.001	4.11	2.06–8.19	<0.001
Tumor type						
Ductal vs. lobular	2.15	0.66–6.95	0.203	2.00	0.47–8.52	0.347
Ductal vs. other	0.89	0.54–1.48	0.664	0.74	0.38–1.44	0.375

## Data Availability

Data from the Cancer Genome Atlas dataset are publicly available at https://www.cancer.gov/about-nci/organization/ccg/research/structural-genomics/tcga (accessed on 19 October 2020). Restrictions apply to the Herlev dataset from Herlev and Gentofte Hospital, Denmark, and are not publicly available under the current research approval from the institutional review board and without a data processing agreement as stated by local law.

## Supplementary Materials

The following are available online at https://www.mdpi.com/article/10.3390/cancers13123050/s1, Figure S1: Flowchart of patients included in the study, Table S1: Clinicopathological characteristics of the patient population.
