# Supplementary material for: Automated Quantification of sTIL Density with H&E-Based Digital Image Analysis Has Prognostic Potential in Triple-Negative Breast Cancers
Source: Cancers (Basel). 2021 Jun 18;13(12):3050. doi: 10.3390/cancers13123050 (PMC8235502; doi:10.3390/cancers13123050)
Supplement: Supplementary file 1 [file cancers-13-03050-s001.zip › cancers-1238977-supplementary.pdf]

cancers

MDPI

Supplementary Material: Automated Quantification of sTIL Density with H&E-Based Digital Image Analysis Has Prognostic Potential in Triple-Negative Breast Cancers

Jeppe Thagaard, Elisabeth Specht Stovgaard, Line Grove Vognsen, Søren Hauberg, Anders B. Dahl, Thomas Ebstrup, Johan Doré, Rikke Egede Vincent, Rikke Karlin Jepsen, Anne Roslind, Iben Kümler, Dorte Nielsen and Eva Balslev

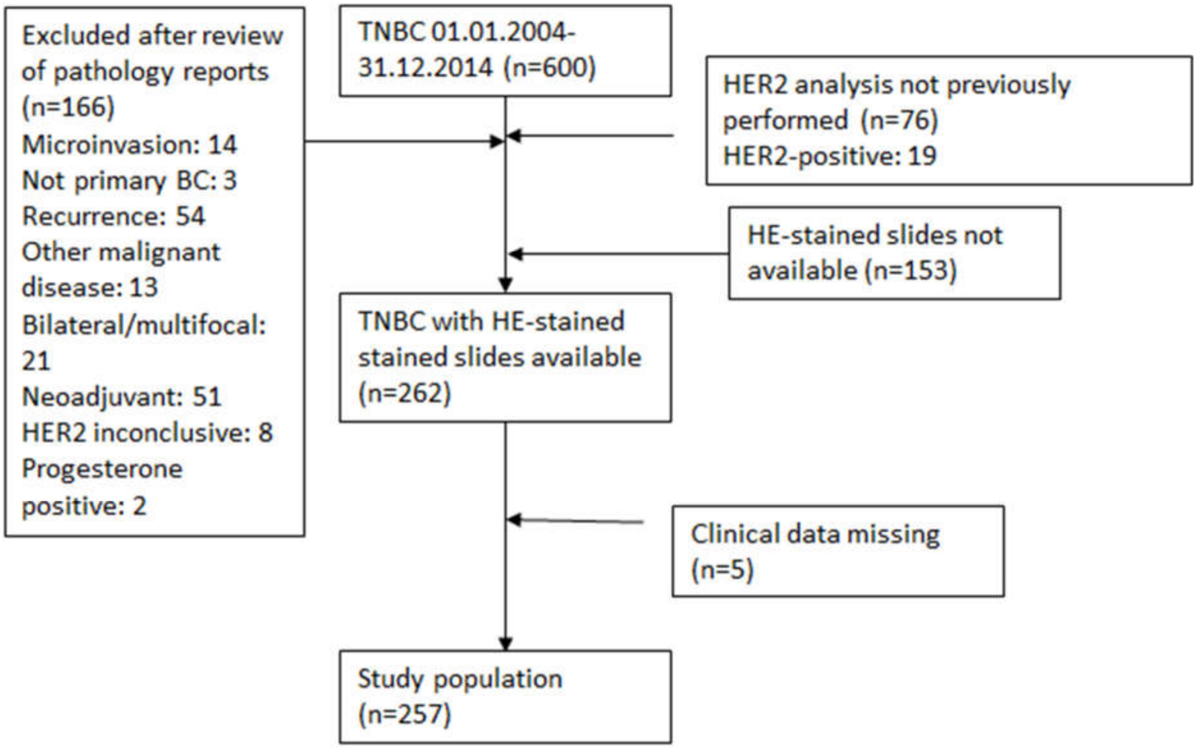

Supplementary Figure S1. Flowchart of patients included in the study.

Supplementary Table S1. Clinicopathological characteristics of the patient population.

**N = 262**<br>**Method**	**Number of patients (%)**
Age	
≤50	66 (25.2)
>50	196 (74.8)
Tumor size	
≤2	108 (41.2)
>2	153 (58.4)
Unknown	1 (0.4)
Tumor type	
Ductal	47 (76)
Lobular	3 (1.1)
Other	60 (22.9)
Nodal status	
0	47 (17.9)
1	18 (6.9)
2	16 (6.1)
Unknown	5 (1.9)
Type of operation	
Mastectomy	100 (38.2)
Lumpectomy	162 (61.8)
